# Estimation of neural voltage traces and associated variables in uncertain models

**DOI:** 10.1186/1471-2202-14-S1-P151

**Published:** 2013-07-08

**Authors:** Pau Closas, Antoni Guillamon

**Affiliations:** 1 Communications Subsystems Area, Centre Tecnològic de Telecomunicacions de Catalunya (CTTC), Av. Carl Friedrich Gauss 7, 08860 Castelldefels, Barcelona, Catalonia, Spain; 2 Departament de Matemàtica Aplicada I, Universitat Politècnica de Catalunya (UPC), Carrer Doctor Marañón 44-50, 08028 Barcelona, Catalonia, Spain

## 

**Figure 1:**
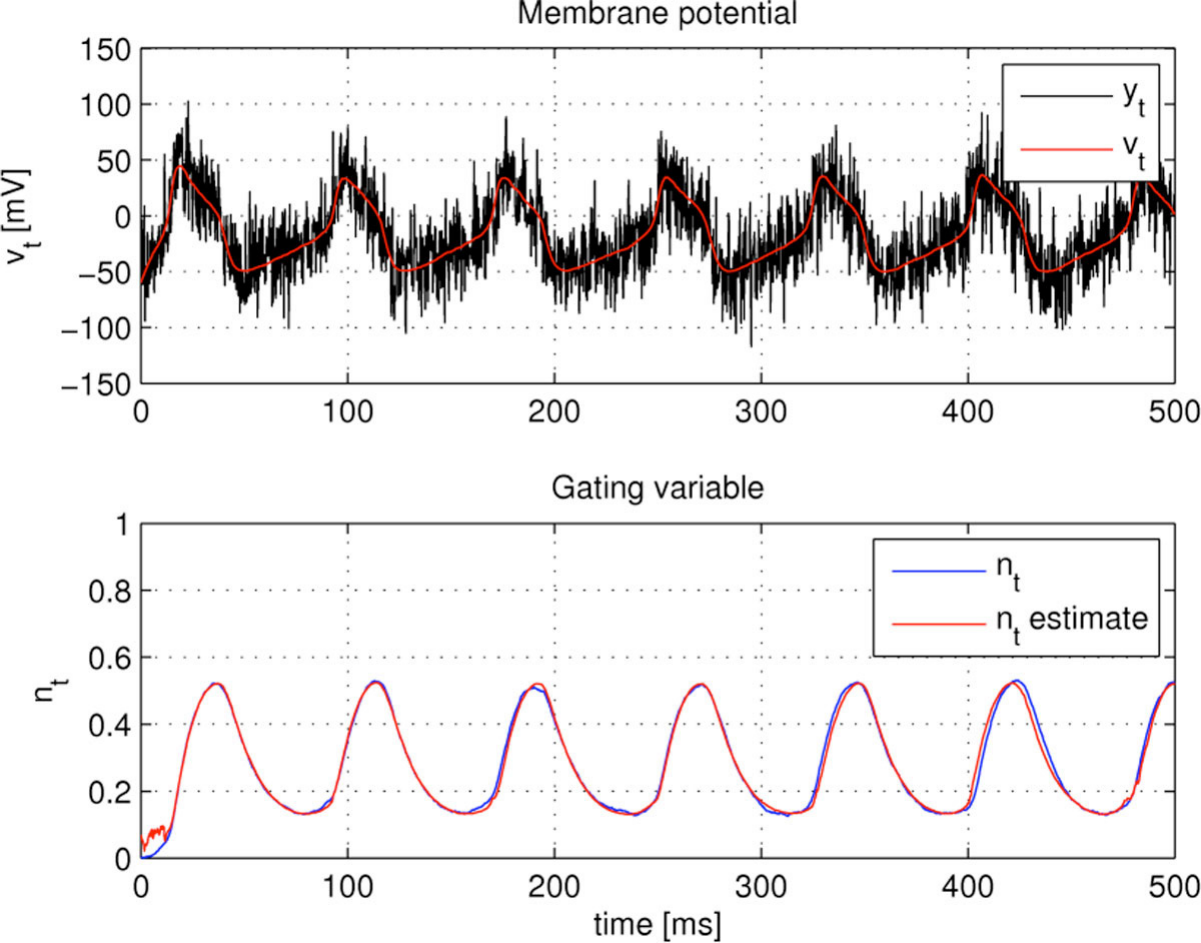
**Top: Noisy membrane potential (y_t_) and estimated time-course (v_t_) with 5 dB of signal-to-noise ratio**. Bottom: time evolution of the true and the estimated K^+ ^gating variable (n_t_) in an uncertain Morris-Lecar model.

Measurements of membrane potential traces constitute the main observables to derive a biophysical neuron model; in particular, the dynamics of auxiliary variables and the model parameters are inferred from voltage traces, in a costly process that typically entails a variety of channel blocks and clamping techniques [1] and some uncertainty in the parameter values due to noise in the signal. Traces are also useful to obtain valuable information about synaptic input, an inverse problem with no satisfactory solution yet (see for instance [2,3]). In this presentation, we are interested in methods that can provide on-line estimation and avoid the need of repetitions that could be contaminated by neuronal variability. Particularly, we concentrate on methods to extract intrinsic activity of ionic channels, namely the probabilities of opening and closing ionic channels. We built a method based on Bayesian theory to sequentially infer these quantities from single-trace, noisy membrane potentials. A dynamical system is assumed for the evolution of the membrane potential and intrinsic variables of the model. Without loss of generality, we considered a Morris-Lecar neuron model to generate simulated data. The model is the a priori information of the Bayesian method, which is efficiently implemented by a particle filter (PF). The method is able to deal with the nonlinear nature of the problem and we observed its robustness to model inaccuracies. The proposed estimation method highly relies on the fact that the neuron model is known. This is true to some extent, but most of the parameters in the model are to be estimated beforehand (this holds for any model). Particularly, we assumed that the applied current, the maximal leakage conductance, and other parameters of the dynamical system were inaccurately known (error of 10%) and model this uncertainty as an increase in the covariance matrix of the model. With the proposed PF with optimal importance density, we recuperated both the membrane potential and the activity of the potassium channel with the minimum attainable error. We derived the theoretical lower bound on the accuracy of any estimator and observed that it is achieved with the presented methodology, therefore the estimator is efficient. In conclusion, we propose a PF that is able to sequentially infer the time-course of the membrane potential and the intrinsic activity of ionic channels from noisy observations of a voltage trace. The results show the validity of the approach and its statistical efficiency (see Figure 1). The procedure can be applied to any neuron model. Forthcoming applications are: combining the presented algorithm with fittings of voltage traces to neuron models; adding synaptic terms to the neuron model and use our method to infer the synaptic conductances. The latter is a challenging topic in the neuroscience literature, where we believe our PF method would give useful results to physiologists that aim at inferring brain's activation rules from neurons' activities.
